# On extending experimental findings to clinical application: Never too late? An advantage on tests of auditory attention extends to late bilinguals

**DOI:** 10.3389/fpsyg.2014.01367

**Published:** 2014-12-03

**Authors:** Andrea A. N. MacLeod

**Affiliations:** École d'orthophonie et d'audiologie, Université de MontréalMontreal, QC, Canada

**Keywords:** bilingual, clinical assessments, vocabulary acquisition, attention, second language learning

In *Never too late? An advantage on tests of auditory attention extends to late bilinguals*, Bak, Vega-Mendoza and Sorace explored whether the bilingual advantage could be observed using a clinical assessment tool of attention, instead of an experimental task. As the authors note, a number of studies have shown a bilingual advantage that extends beyond the linguistic realm to cognitive tasks. In particular, bilingual children and adults have been shown to outperform monolingual peers on executive function tasks such as the control of attention (Bialystok and Martin, 2004; Bialystok et al., 2004). In addition to better performance on these experimental tasks, older bilingual adults have been reported to show a 4–5 years delay in the onset of dementia when compared to older monolingual adults (Bialystok et al., 2007). Although a bilingual advantage has been observed in experimental tasks, it is less clear if they would show this advantage on clinical tasks of attention control. Bak and his colleagues demonstrate that bilingual adults maintain this advantage on the auditory attention subtests of the Test of Everyday Attention. Two key issues should be considered in future research: first, we need to consider the clinical implications of these results; and second, we need to carefully describe bilingual participants to allow for the application of research findings to clinical practice. In exploring these issues, I will draw parallels with research in the assessment of vocabulary among bilingual children.

The application of clinical assessment tools to bilingual populations is a critical step in the fields of speech-language pathology and clinical psychology. In language assessments, particularly those used to assess children, the clinical tools tend to underestimate the children's language capacities (Umbel et al., 1992; Pearson, 1998; Bialystok et al., 2010) for two main reasons. First, bilingual children are often assessed in only one of their languages (Caesar and Kohler, 2007), and thus strengths in the other language are not documented. Second, the assessment tools have been developed and normalized on monolingual children, and thus the bilingual's score may be typical for a bilingual child, but not for a child acquiring a single language (Bedore et al., 2005). The case of vocabulary assessments provides a simple illustration of the downfalls of using tasks developed for monolingual populations. Bilingual children do not have identical lexical knowledge in both of their languages: they have a shared vocabulary (e.g., knowledge of the word for “tree” in two languages), and language specific vocabulary (e.g., knowledge of the word “multiplication” in the language used at school, and of “house coat” in the language used at home). Bilingual children often score lower than their monolingual peers in each of their languages (Pearson et al., 1993; Core et al., 2013), which can result in a referral to a speech-language pathologist for treatment. The referral may not be appropriate, even if the child is assessed in both languages, since the assessment tool does not account for the bilingual child's shared and language specific vocabulary (Bedore et al., 2005). A better assessment of vocabulary would consider the child's lexical knowledge using either a measure of total vocabulary (all words known), or conceptual vocabulary (concepts known regardless of language). Researchers have demonstrated that typically developing bilingual children score the same or higher than their monolingual peers when measured using their total vocabulary (Core et al., 2013), and conceptual vocabulary (Bedore et al., 2005). In the absence of a clinical tool that can accurately measure the bilinguals' vocabulary, it is important to develop normative data based on bilingual children.

In the present study, Bak and his colleagues have shown that the clinical tool, the Test of Everyday Attention, is sensitive to differences between bilingual and monolingual adults. In contrast to the vocabulary example above, the bilingual adults scored higher than their monolingual peers on the auditory subtests. From a clinical point of view, a higher score may not seem problematic, since it would not lead to a referral for further assessment or intervention. Instead, perhaps the criteria for referral for a bilingual patient should be adjusted upward in light of the higher performance of typical bilingual adults. For example, a bilingual patient who scored lower than his bilingual peers following a stroke may still be within the range of “normal” for a monolingual adult: should this lower score relative to his bilingual peers lead to more in depth evaluation? An important next step in this line of research would be to address the clinical significance of the difference observed. In particular, do bilingual adults who suffer from neurological traumas (e.g., stroke or head injury) or neurodegenerative conditions maintain the bilingual advantage?

In taking this next step, it will be important to carefully describe the bilingual population under study. As Bak and his colleagues found, bilingual adults may perform differently due to differences in age of second language acquisition: in comparison to monolinguals, adults who acquired two languages during early childhood performed better on the attention switching task, and those who acquired their L2 during late childhood and adolescence performed better on the selective attention task. Other research has shown that bilingual may also differ due to the contexts of second language learning, the contexts of on-going language use, and their abilities across different modalities. For example, in a study of bilingual children's vocabulary development in French and German, the amount of exposure played a strong role in the children's vocabulary development, despite simultaneous acquisition of both languages and daily exposure to both languages (MacLeod et al., 2013). In a series of studies that focused on bilinguals who spoke Welsh and English, Gathercole and her colleagues have documented the complex interplay between age of second language exposure, language learning context (home, school, or both), and on-going language use (Gathercole and Thomas, 2009; Gathercole et al., 2010). For example, in their study of executive function tasks among bilingual children, they found a bilingual advantage for bilingual children living in homes that used only Welsh (Gathercole et al., 2010). In addition to the context of language use, the bilinguals with more balanced use of both languages showed a stronger advantage (Gathercole et al., 2010). In experimental studies, a careful description of bilingual participants allows for replication and comparison across studies. This careful description is particularly important when applying findings to a clinical setting in order to provide the most accurate and appropriate services to patients.

I concur with Bak, Vega-Mendoza and Sorace: it is important to evaluate whether clinical tools are sensitive to bilingual abilities. Future research needs to carefully describe the bilingual participants with regards to age of second language acquisition, but also the context of language learning and on-going language use. Of equal importance is pursuing this line of research to understand whether the differences observed are clinically important. In the case of the assessment of bilingual children's vocabulary, the clinical impact was clear: bilingual children were at risk of over-diagnosis of a language disorder. For the control of attention, the clinical significance of the results remains to be documented but may result in the development of new criteria on attention subtests for bilingual adults.

## Conflict of interest statement

The author declares that the research was conducted in the absence of any commercial or financial relationships that could be construed as a potential conflict of interest.
